# Epitope design of L1 protein for vaccine production against Human Papilloma Virus types 16 and 18

**DOI:** 10.6026/97320630013086

**Published:** 2017-03-31

**Authors:** Sunanda Baidya, Rasel Das, Md. Golam Kabir, Md. Arifuzzaman

**Affiliations:** 1 Department of Biochemistry & Molecular Biology, University of Chittagong, Chittagong 4331, Bangladesh;; 2 Leibniz Institute for Surface Modification, Permoserstraße 15, 04318 Leipzig, Germany;; 3 Department of Biochemistry and Biotechnology, University of Science and Technology Chittagong (USTC), Foy’s Lake, Chittagong 4202, Bangladesh;

**Keywords:** Human Papilloma Virus, capsid proteins, cervical cancer

## Abstract

Cervical cancer accounts for about two-thirds of all cancer cases linked etiologically to Human Papilloma Virus (HPV). 15 oncogenic HPV types can cause cervical cancer, of which HPV16 and HPV18 combinedly account for about 70% of it. So, effective epitope design for the clinically relevant HPV types 16 and 18 would be of major medical benefit. Here, a comprehensive analysis is carried out to predict the epitopes against HPV types 16 and 18 through “reverse vaccinology” approach. We attempted to identify the evolutionarily conserved regions of major capsid protein (L1) as well as minor capsid protein (L2) of HPV and designed epitopes within these regions. In this study, we analyzed about 49 and 27 sequences of HPV L2 and L1 proteins respectively. Since we found that the intertype variability of L2 is higher than for L1 proteins, our analysis was emphasized on epitopes of L1 of HPV types 16 and 18. We had selected HLA-A*0201, DRB1*1501, DQB1*0602, DRB1*0401 and DQB1*0301 alleles for the prediction of T cell epitopes of L1 of HPV 16 and 18. Finally, we reported that predicted epitope sequences EEYDLQFIFQLCKITLTA, and RHGEEYDLQFIFQLCKITLTA of L1 protein of HPV 16, and LPDPNKF, PETQRLVWAC, PVPGQYDA, YNPETQRLVWAC, DTGYGAMD, PVPGQYDATK, KQDIPKVSAYQYRVFRV, RDNVSVDYKQTQLCI and YSRHVEEYDLQFIF of L1 protein of HPV 18 could be therapeutic tools for vaccine design against HPV.

## Background

Human papillomaviruses (HPVs) are small in shape, nonenveloped, double stranded DNA viruses whose eight proteins are encoded by approximately 8 kb circular genome [1,2]. The HPV genome can be classified into three regions; the long control region (LCR), the early open reading frame (EORF) and the late reading frame (LORF) [3]. The viral ORFs are comprised of three regulatory genes involved in transcription and replication (E1, E2, and E4), three oncogenes (E5, E6, and E7) and two genes encoding for self-assembling proteins, which constitute the viral capsid (L1 and L2) [4]. To date, this virus is reported to cause 99% of cervical cancer [5], 93% of anus [6], 40% of vagina [7], 40% of penis [8], 63% of oropharynx [9] and 51% of vulva cancers [10]. Moreover, it is estimated that every year about 527,600 women are diagnosed with cervical cancer and 265,700 die from the disease [11]. To fight with HPV infections, the developed countries have increased financial supports to improve HPV vaccination, for example, USA have allocated health budget of $4 billion/year in this regard [12]. For developing countries, prevention of this viral infection is of more importance, since it contributes about 85% to global cervical cancer burden [4].

Among 15 oncogenic HPV types (16, 18, 31, 33, 35, 39, 45, 51, 52, 56, 58, 59, 68, 73, and 82), HPV 16 and HPV 18 are most dominant, accounting for about 50% and 20% of cervical cancer respectively [13]. Cumulatively these 2 types also account for an even higher proportion of other genital and mucosal cancers attributable to HPV infection [14]. Therefore, prevention can be introduced by inhibiting them either through vaccination or postinfective treatments. Vaccination is potentially the hardest currency to prevent viral infections in both pediatric and adolescent phase [15]. HPV capsid proteins L1 and L2 can provide tremendous potentiality for the vaccine design. Currently, two prophylactic vaccines, Merck’s Gardasil and GlaxoSmithKline’s Cervarix, based on L1 antigen have been approved and implemented in more than 100 countries [16,17]. Although both vaccines are highly effective in preventing HPV infections, still neither one show therapeutic effects to established HPV infections. Also, administration of these vaccines has been found to have severe side effects, whereas the therapeutic vaccine is considered to be safe, stable and easily producible [18]. Different therapeutic strategies have emerged including vectorbased, peptide-based, protein based, DNA based, cell based and combinational approaches. Here we have focused on the prediction of epitopes for peptide based therapeutic vaccination against HPV 16 and 18.

The availability of genomic data, understanding of immune responses and immunogenetic variations, new developments in computer applications and systems biology approach are showing new directions in vaccine development, away from the traditional techniques of live-attenuated or inactivated virus or recombinant VLP vaccines. The primary goal of this study is to investigate different conserved epitopes of L1 and L2 capsid proteins through ‘’Reverse Vaccinology’’ approach [19] and justify whether they are immunogenic or not. The field of immunoinformatics, which deals with the mapping of B-cell and T-cell epitopes [20] has convinced researchers to look further in search of epitopes which not only minimizes the number of experiments but also enables a systematic identification of candidate epitopes [21] from which experimental testing could be made easier. The possibility of provoking any reaction against self-antigens is also reduced by peptide-based vaccines, thereby proving to be a safer vaccine by inhibiting the stimulation of auto immunity [22]. Since epitopes are regions present on the antigens, which are easily recognized by the antibodies [23], epitope-based techniques provide the accurate and precise characterization of Immune responses [24]. Moreover, the fundamental purpose of epitope prediction is to design a molecule that can mimic a genuine epitope’s structure and function and replace it in medical diagnostics, therapeutics and vaccine design [25]. In addition to all these, the most important and prior advantage of epitope-based vaccines is that it deletes the possibility of using the whole deadly viral proteins [26]. Thus prediction of reliable epitopes is essential for rational vaccine design, which can identify the potential targets, and inturn paves way for immunotherapeutic cancer treatment [27].

The peptide-based vaccine also has another advantage of increased safety, the opportunity to rationally engineered epitopes for increased potency and the ability to focus immune responses on conserved epitopes [28,29]. Thus, the aim of our study was to identify B-cell and T cell epitopes of the L1 and L2 proteins of the two high risk HPVs (HPV 16 and HPV 18). Since multiple sequence alignment results revealed higher intertype variability of the L2 protein than L1 and experimental validation provided no presence of conserved epitope in L2, We focused our analysis on L1. Finally in this work we have found some linear epitopes, which can defined as consist of conserved amino acids and are easier to express and mimic than conformational epitopes [30,31], EEYDLQFIFQLCKITLTA, RHGEEYDLQFIFQLCKITLTA, LPDPNKF, PETQRLVWAC, PVPGQYDA, YNPETQRLVWAC, DTGYGAMD, PVPGQYDATK, KQDIPKVSAYQYRVFRV, RDNVSVDYKQTQLCI and YSRHVEEYDLQFIF of L1 protein, which can be used for vaccine design against HPV, especially HPV 16 and 18.

## Methodology

### Sequence alignment

Amino acid sequences of the capsid proteins (L1 and L2) of HPV types were retrieved from the NCBI database available at (http://www.ncbi.nlm.nih.gov/) updated till December 24, 2016 and the accession numbers of those sequences are listed in Table 1. Conserved regions in L1 and L2 capsid protein sequences of human papilloma virus (HPV) were determined by using nucleotide sequence alignment software database (CLUSTALW) program available at (http://www.genome.jp/tools/clustalw/) [32].

### Epitope prediction

B cell epitopes of L1 and L2 protein sequences of HPV were predicted by using Immune Epitope Database (IEDB) Analysis Resource, available at (http://tools.immuneepitope.org/main /html/bcell_tools.html) [33]. Kolaskar & Tongaonkar Antigenicity method used to predict all potential epitopes with standard features [34].

T cell epitopes of L1 and L2 protein sequences of HPV were collected by T Cell Epitopes- MHC Binding Prediction Tools by using Immune Epitope Database (IEDB) web tool available at (http://tools.immuneepitope.org/). Those epitopes, who have potentiality to bind with MHC class I molecule were determined by peptide binding to MHC Class I molecules Tool [35-38]. In addition, epitopes binding to MHC class II molecule were determined by peptide binding to MHC Class II molecules Tool [39,40].

### Epitope locomotion

The 3D structures of L1 protein of HPV (types 16 and 18) were retrieved from the Protein Data Bank (PDB) with folling IDs 2R5H and 2R5I, respectively available at (http: www.pdb.org/). The PDB ID (2R5H) and (2R5I) were used at MMDB (Molecular Modeling Database) at (http://www.ncbi.nlm.nih.gov/Structure/MMDB/mmdb.shtml) [41] to determine 3D structures of epitopes. The PyMOL software (https://www.pymol.org) was then utilized to see the location of linear epitopes in 3D structures of L1 protein of HPV types 16 and 18.

## Result

### Alignment of amino acid sequences of capsid proteins (L1 and L2) of human papilloma virus

HPV capsid proteins L1 and L2 usually synthesize late in the infection cycle and encapsidate the closed circular doublestranded DNA mini-chromosome. Since L1 and L2 capsid proteins are largely responsible for essential functions including virion assembly, virion stability, and virion uncoating, a consistent pattern of conserved and variable regions is perhaps not surprising. We had aligned 27 sequences of L1 and 49 sequences of L2 proteins of HPV obtained from the National Center for Biotechnology Information database (http://ncbi.nlm.nih.gov) by using CLUSTALW program to find conserved regions with in the sequences. This is because the highly conserved epitopic regions are less prone to antigen escape and viral mutation [26]. From the alignment figures (not shown here) we found that the intertype variability of the L2 proteins is higher than for the L1 proteins. Thus, L2 proteins show far less consistency in the positions of variable regions than is seen in the L1 proteins.

### B cell and T cell epitopes of L1 and L2 proteins of human papilloma virus (HPV)

To predict continuous linear B cell epitopes we used Kolaskar & Tongaonkar Antigenicity Method [34]. This method is a semiempirical method that makes use of physicochemical properties of amino acid residues and their frequencies of occurrence in experimentally known segmental epitopes was developed to predict antigenic determinants on proteins [34]. Application of this method to a large number of proteins has shown that the method can predict antigenic determinants with about 75% accuracy which is better than most of the known methods. According to Kolaskar & Tongaonkar the greater the score of an epitope, the greater the antigenicity. We have found B cell epitopes with greater score in the conserved regions within the sequences of L1 protein of HPV type 16 and type 18 but no potential B cell epitope had been found in the conserved regions of L2 protein sequences.

Here T cell MHC-I epitopes were predicted by using Peptide binding to MHC class I molecules tool [Immune Epitope Database (IEDB)]. This tool considers in an amino acid sequence or set of sequences and determines each subsequence's ability to bind to a specific MHC class I molecule. Consistent dysregulation of HLA expression has been reported in cervical neoplasia, which can influence the prospects for cell-mediated vaccine therapies. HLA-A*0201 allele’s expression has been found to be increased in HPV-associated cervical cancer [42]. Therefore, we selected HLAA* 0201 allele for prediction of T cell MHC-I epitopes of L1 and L2 proteins of HPV. In this study some potential T cell MHC-I epitopes were found in conserved regions within the sequences of L1 protein of HPV type 16 and 18. No T cell MHC-I epitope was found in conserved regions of HPV L2 protein sequences.

T cell MHC-II epitopes were predicted by using Peptide binding to MHC class II molecules tool [Immune Epitope Database (IEDB)]. This tool provides a consensus approach to predict MHC Class II epitopes based upon Sturniolo, ARB, and SMM align. Several HLA class II alleles have been found to increase the risk of cervical cancer when associate with it [43,44]. DRB1*1501 and DQB1*0602 alleles were found to be associated only with HPV 16 possitive cervical cancer [45]. Thus, we selected HLA- DRB1*1501 and DQB1*0602 alleles to predict T cell MHC-II epitopes of L1 protein of HPV 16. The most important at-risk alleles are DQB1*0301 and DRB1*0401, when associated with HPV 18 positive cancers [46]. So we had selected DRB1*0401 and DQB1*0301 to predict T cell MHC-II epitopes of L1 protein of HPV 18. Some potential T cell MHC-II epitopes of L1 proteins of HPV 16 and 18 were found but no one was found in conserved regions within the sequences of L2 protein of HPV.

### B cell and T cell linear epitopes of L1 protein of human papilloma virus (HPV) types 16 and 18

**Figure 1:**
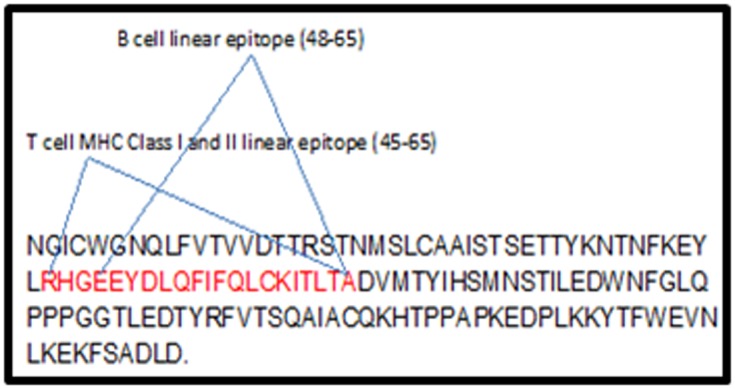
Sequence diagram of HPV 16 L1 protein. Sequence diagram of HPV 16 L1 protein. Here epitope sequences and their locations (amino acid) in L1 protein are highlighted in red and blue respectively.

**Figure 2:**
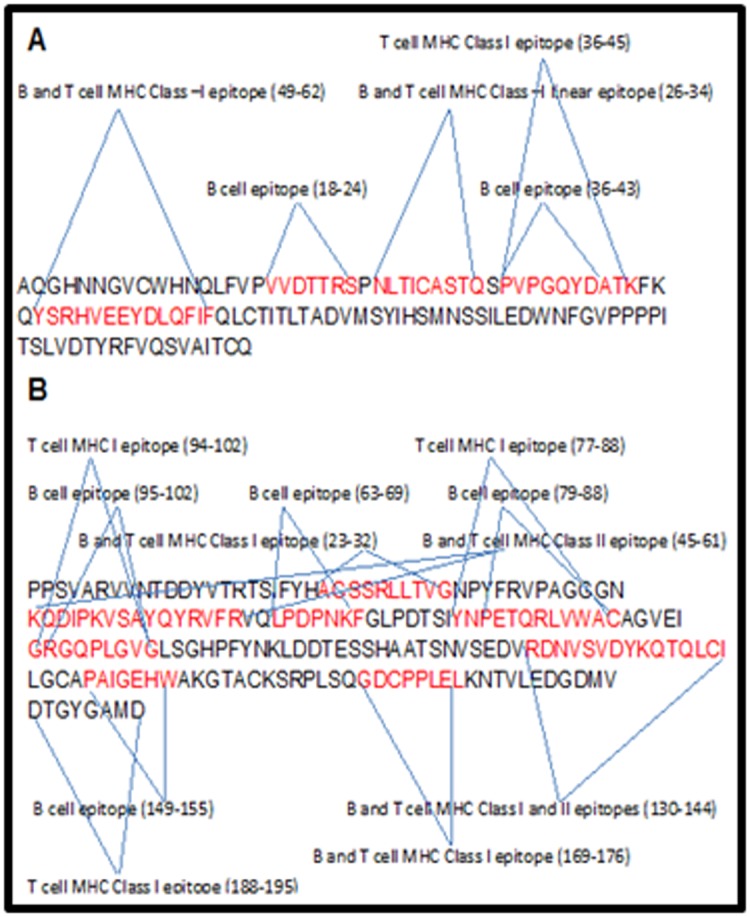
Sequence diagrams of HPV 18 L1 protein (A and B). (A): Sequence diagram of HPV 18 L1 protein. Here all are linear epitopes and their sequences and locations (amino acid) in L1 protein are highlighted in red and blue respectively. (B) Sequence diagram of HPV 18 L1 protein. Here all are linear epitopes and their sequences and locations (amino acid) in L1 protein are highlighted in red and blue respectively.

Vaccines composed of longer peptide sequences generate both CD4 and CD8 specific T cell responses against HPV oncogenes. It results in more vigorous CD8 CTL responses than vaccination with exact minimal CTL (Cytotoxic T lymphocytes) epitope length [47]. We had chosen B and T cell epitopes with longer length due to their high potentiality to stimulate immune system. We selected B cell linear epitope EEYDLQFIFQLCKITLTA and T cell MHC Class-I and II epitope RHGEEYDLQFIFQLCKITLTA whose locations in L1 protein sequence of HPV 16 have been shown in Figure 1. We also selected B cell linear epitopes VVDTTRS, PVPGQYDA, LPDPNKF, PETQRLVWAC, RGQPLGVG and PAIGEHW; T cell MHC Class –I linear epitopes PVPGQYDATK, GRGQPLGVG, YNPETQRLVWAC and DTGYGAMD; B cell and T cell MHC Class –I linear epitopes AGSSRLLTVG, NLTICASTQ, YSRHVEEYDLQFIF and GDCPPLEL; B cell and T cell MHC Class –II linear epitope KQDIPKVSAYQYRVFRV; B cell and T cell MHC Class –I and II linear epitopes RDNVSVDYKQTQLCI Whose locations in L1 protein sequences of HPV 18 have been shown in (Figure 2A and 2B). All the aforementioned linear epitopes, observed in most conserved regions in sequences of L1 protein of HPV type 16 and 18.

**Figure 3:**
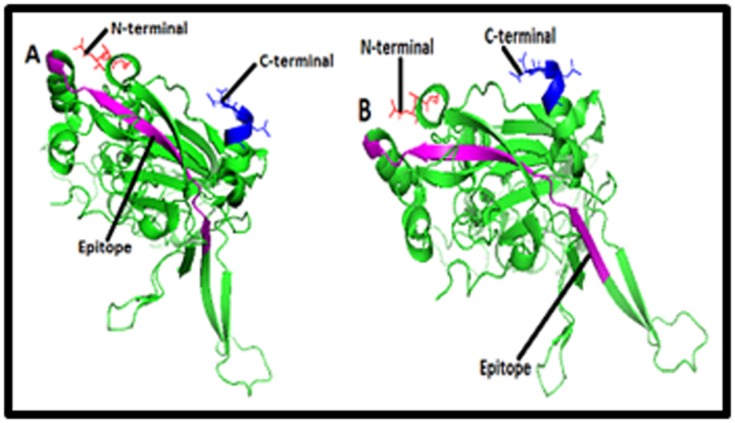
Structures of (A) B cell epitope [EEYDLQFIFQLCKI TLTA] and (B) T cell epitope [RHGEEYDLQFIFQLCKITLTA] in the cartoon of HPV type 16 L1 protein. Structures of B cell epitope [EEYDLQFIFQLCKITLTA] and (B) T cell epitope [RHGEEYDLQFIFQLCKITLTA] in the cartoon of HPV type 16 L1 protein are highlighted here in purple color. HPV 16 L1 is a pentameric protein which has A, B, C, D, E, F, G, H, I, J, K, L, M, N and O chains. Here only 3D structure of ‘’A’’ chain has been shown and in the structure red and blue color represent the amino and carboxyl terminals respectively.

**Figure 4:**
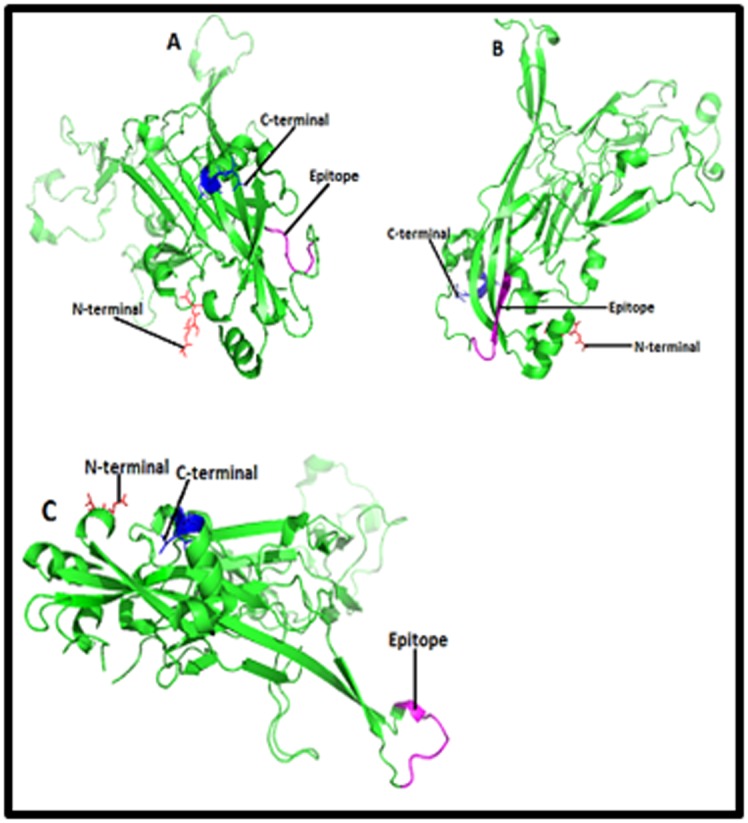
Structures of B cell epitopes (A) [LPDPNKF], (B) [PETQRLVWAC] and (C) [PVPGQYDA] in the cartoon of HPV type 18 L1 protein. Structures of B cell epitopes (A) [LPDPNKF], (B) [PETQRLVWAC] and (C) [PVPGQYDA] in the cartoon of HPV type 18 L1 protein are highlighted here in purple color. HPV 18 L1 is a pentameric protein which has A, B, C, D, E, F, G, H, I, J, K, L, M and N chains. Here only 3D structure of ‘’A’’ chain has been shown and in the structure red and blue color represent the amino and carboxyl terminals respectively.

**Figure 5:**
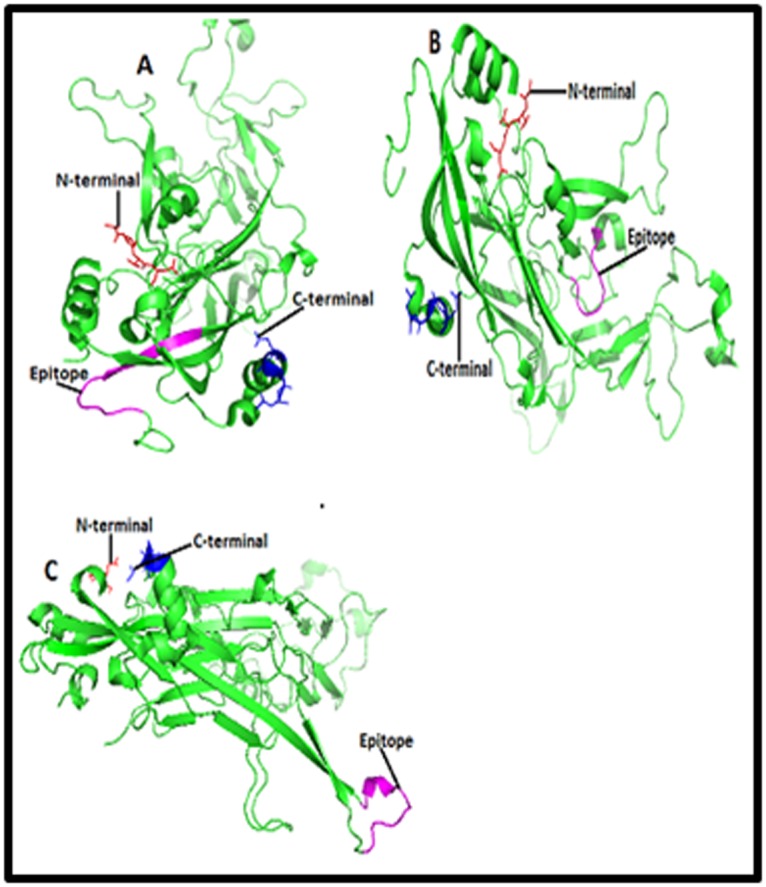
Structures of T cell epitopes (A) [YNPETQRLVWAC], (B) [DTGYGAMD] and (C) [PVPGQYDATK] in the cartoon of HPV type 18 L1 protein. Structures of T cell epitopes (A) [YNPETQRLVWAC], (B) [DTGYGAMD] and (C) [PVPGQYDATK] in the cartoon of HPV type 18 L1 protein are highlighted here in purple color. HPV 18 L1 is a pentameric protein which has A, B, C, D, E, F, G, H, I, J, K, L, M and N chains. Here only 3D structure of ‘’A’’ chain has been shown and in the structure red and blue color represent the amino and carboxyl terminals respectively.

**Figure 6:**
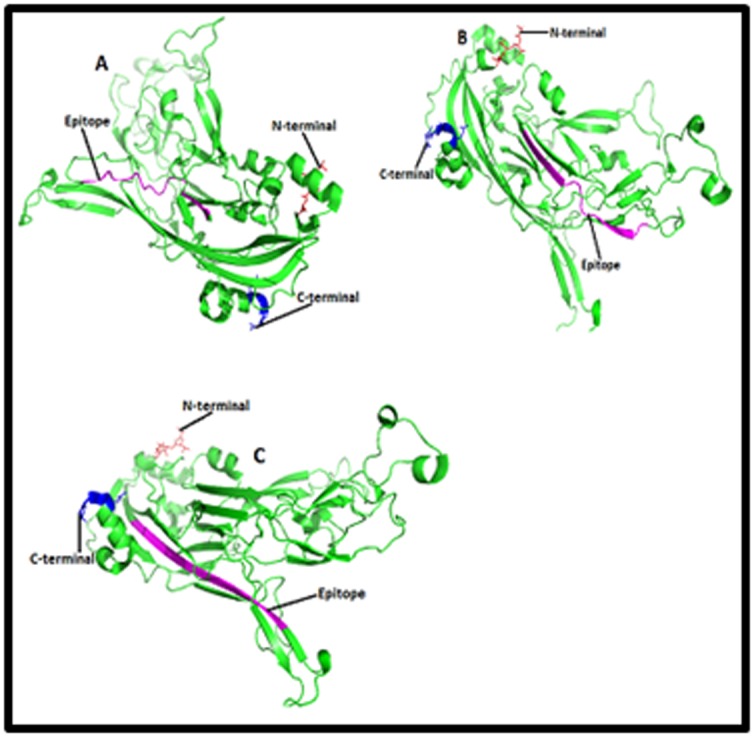
Structures of B and T cell epitopes (A) [KQDIPKVSAYQYRVFRV], (B) [RDNVSVDYKQTQLCI] and (C) [YSRHVEEYDLQFIF] in the cartoon of HPV type 18 L1 protein. Structures of B and T cell epitopes (A) [KQDIPKVSAYQYRVFRV], (B) [RDNVSVDYKQTQLCI] and (C) [YSRHVEEYDLQFIF] in the cartoon of HPV type 18 L1 protein are highlighted here in purple color. HPV 18 L1 is a pentameric protein which has A, B, C, D, E, F, G, H, I, J, K, L, M and N chains. Here only 3D structure of ‘’A’’ chain has been shown and in the structure red and blue color represent the amino and carboxyl terminals respectively.

Furthermore, three dimensional structure of L1 protein of HPV type 16 and type 18 were retrieved by using PDB (Protein Databank) ID 2r5h and 2r5i respectively (Figure 3, Figure 4, Figure 5and Figure 6).

### Location of B cell and T cell linear epitopes

Finally we had choosen those B and T cell linear epitopes who have aromatic amino acids [phenylalanine (F), tryptophan (W) and tyrosine (Y)], as aromatic amino acids have been found to increase immunogenecity. Epitope sequences are shown in purple color in the Figure 3, Figure 4, Figure 5and Figure 6.

Epitopes 48EEYDLQFIFQLCKITLTA65, and 45RHGEEY DLQFIFQLCKITLTA65 of L1 protein of HPV type 16, Figure 3 and epitopes 63LPDPNKF69, 79PETQRLVWAC88, 36PVP GQYDA43, 77YNPETQRLVWAC88, 188DTGYGAMD195, 36P VPGQYDATK45, 45KQDIPKVSAYQYRVFRV61, 130RDNV SVDYKQTQLCI144 and 49YSRHVEEYDLQFIF62 of L1 protein of HPV type 18, Figure 4, Figure 5and Figure 6 were predicted as potential epitopes and superscript numbers indicate the position of epitope in the L1 protein sequence.

## Discussion

Worldwide HPV-induced dysplasia and cancer cause significant morbidity [48]. Although prophylactic vaccines are presently accessible, immunization does not reach everyone at risk. Moreover, those vaccines have no therapeutic effects [49], leaving HPV-infected individuals in need of treatment options. A noninvasive treatment such as a therapeutic vaccine employing an effective anti-HPV state would be an attractive alternative. Still now prediction of immunogenic epitopes for HPV is a vital and challenging task. Though there are various types of high risk HPVs, HPV 16 and HPV 18 are responsible for 70% of cervical adenocarcinomas [50]. In this work we have made an attempt to predict the epitopes of the L1 proteins of high risk HPVs (16 and 18). To achieve this task, we analyzed a huge amount of data and arrive at an interesting result. Our work focused on identifying the conserved residues, T cell (HLA class I and HLA class II) and B cell epitopes and their corresponding 3D structure information.

From the multiple sequence alignment results, we found that the intertype variability of the L2 proteins is higher than L1. Thus, L2 is less consistent in the positions of variable regions than the L1. Conserved regions of L1 and L2 revealed that more epitopes present in conserved regions within the sequences of L1 than L2 protein and these regions would be major targets for neutralizing antibodies. Thus, L1 protein could be a better target for vaccine design than L2. We preferentially selected B cell and T cell linear epitopes with longer length because vaccination with longer peptides generates high levels of neutralizing antibodies than vaccination with exact minimal epitope length [47]. Several HLA class I and class II alleles associate with the risk of cervical cancer. Here, we selected HLA-A*0201 allele for prediction of T cell MHC-I epitopes of L1 Protein of HPV 16 and 18. The DRB1*1501 and DQB1*0602 alleles and the HLA- DRB1*1501 and DQB1*0602 alleles were found to be associated only with HPV 16 and HPV 18 positive cervical cancer respectively [45,46]. Thus, HLADRB1* 1501 and DQB1*0602 alleles were selected to predict T cell MHC-II epitopes of L1 of HPV 16 and DRB1*0401 and DQB1*0301 alleles to predict T cell MHC-II epitopes of L1 protein of HPV 18. Since the location of epitopes are critical for the production of neutralizing antibodies [51,52], we predicted three-dimensional model to further elucidate probable aspects of those epitopes of L1 protein, who have aromatic amino acids [phenylalanine (F), tryptophan (W) and tyrosine (Y)], as presence of aromatic amino acids increase immunogenecity.

Finally, we hypothesized that epitopes 48EEYDLQFIFQLCK ITLTA65, and 45RHGEEYDLQFIFQLCKIT LTA65 Figure 3 of L1 protein of HPV type 16, and epitopes 63LPDPNKF69, 79PETQRLVWAC88, 36PVPGQYDA43, 77YNPETQRLVWAC88, 188DTGYGAMD195, 36PVPGQYDAT K45, 45KQDIPK VSAYQYRVFRV61, 130RDNVSVDYKQTQ LCI144 and 49YSRHVEEYDLQFIF62 Figure 4, Figure 5and Figure 6 of L1 protein of HPV type 18 might be the epitopes associated with production of neutralizing antibody response against HPV type 16 and 18, since they follow all the factors that influence immunogenicity. Immunogenicity of an immunogen can be determined by some properties: its foreignness, molecular size, chemical nature, complexity, heterogenicity and ability to be processed and presented with an MHC molecule. Epitopes of HPV L1 proteins are foreign particles for human. The molecular weight of HPV L1 protein is 55 kDa, and the most active immunogen tend to have a molecular mass of > 100,000 Daltons (Da), so epitopes of L1 protein could be effective immunogen. Proteins found to have greater

Immunogenicity than carbohydrates, lipids and nucleic acids and here we had designed epitopes of a protein. Primary structure of proteins found to have lesser immunogenicity than secondary structure of same protein. Since epitopes of HPV L1 protein were shown in 3D structures they could be potential immunogen. Heteropolymers (composed of different amino acids) are more immunogenic than homopolymers. Since epitopes of HPV L1 protein are heteropolymer, they could be effective immunogen.

Several studies had been reported some epitopes of HPV L1 protein such as epitope sequences MVLILCCTLAILFCVA, LAILFCVADVNVFHIF, DVNVFHIFLQMSVWRP and QMSVWRPSEATVYLPP of HPV type 58 [53]; epitopes ENVPDDLYIKGSGS, QPLGVGISGHPLLNKLDDTE, GLKA KPKFTLGKRKATPTT, NLASSNYFPTPSGSM, PSGSM VTSDAQIFNK, STILEDWNFGLQPPPGGTLE, PCTN VAVNPGDC, FNRAGTVGENVPDDLYIKGS and DVNV YHIFFQMSLWLPSEAT of HPV type 16 [54-58]; epitopes STILEDWNFGLQPPPGGTLE, SSILEDWNFGVPPPPTTSLV and QPLGVGISGHPLLNKLDDTE of HPV type 18 [54-57]; epitopes STILEDWNFGLQPPPGGTLE and QPLGVGISGHPLLNKLDDTE of HPV types 31 and 11 [54-57] and epitope QPLGVGISGHPLLNKLDDTE of HPV type 33, 35 and 45 [54]. Subramanian and Chinnappan, 2013 [59]; reported that the fragments FAFK(R) DL and KLPD(Q)LCTEL are most promiscuous epitopes among E6 proteins of high risk HPVs - HPV 16, HPV 18 and HPV 45 by the application of “Reverse Vaccinology’’ similar to our study. Here we predicted potential epitopes of L1 proteins of HPV 16 and HPV 18 instead of E6 proteins of HPV 16, 18 and 45. Dey et al. 2016 [60]; presented a study for the designing of multivalent peptide vaccines against multiple HPV types and reported both linear and conformational epitopes of L1 proteins of HPV 16, 18, 33, 35, 45, and 11 types. The caveat to our analysis is that we had only predicted linear epitopes in conserved regions of HPV 16 and 18. Combita et al. 2002 [54]; experimenting with mice model confirmed that linear epitopes induce cross-neutralization, but also inferred that such cross-neutralization will not exceed 1% of the effects of the dominant conformational epitopes. Rodden et al. 2000 [61]; reported that type-specific and cross-reactive linear epitopes have been determined on the L2 protein surface also, but in this study, we restrict our analyses to the L1 protein epitopes only as we did not found any linear epitopes in the conserved regions of L2. One potential caveat of our current analysis is that the developed immunity will be HPV type-specific so that protection will occur only to HPV 16 and 18 types. As HPV-associated cancers are comprised of at least 15 HPV types, future experiments will be required for the designation of a multi-HPV-type vaccine, or a vaccine that develops more broadly protective neutralizing Abs, for more complete protection against HPV-associated cancers.

## Conclusion

Therapeutic vaccines play a vital role in preventing the metastatic spread of cervical cancer with immediate impact rather than prophylactic vaccines, which normally takes many years to reduce deaths from this disease. We have proposed an alternative strategy of peptide vaccines for conditions where VLP (Virus like Particle) vaccines like Gardasil and Cervarix may not be ideal. Our exercise yielded a set of epitopes of L1 protein for HPV type 16 and 18. Because of the nature of the viral coat proteins, the epitopes proposed in this paper may not provide the high level of efficiency that the VLP vaccines can provide, but with suitable adjuvants may enhance the neutralizing antibody production. Hence, we conclude that, epitopes 48EEYDLQFIFQLCKITLTA65, 45RHGEEYDLQFIFQLCKITLTA65, 63LPDPNKF69, 79PETQRLV WAC88, 36PVPGQYDA43, 77YNPETQRLVWAC88, 188DT GYGAMD195, 36PVPGQYDATK45, 45KQDIPKVSAYQY RVFRV61, 130RDNVSVDYKQTQLCI144 and 49YSRHVEEY DLQFIF62 of L1 protein might be used as potential tools for designation of a new vaccine against HPV type 16 and 18. However, it is necessary to perform wet laboratory experiments to test the efficacy of the results of studies like this. At last such preparations should be strongly recommended due to the wide spread of papillomavirus-induced cervical, genital, and other cancers and the sufferings these cause.

## Figures and Tables

**Table 1:** Accession numbers of sequences of HPV L2 and L1 proteins.

HPV Types	HPV Proteins	Accession Numbers
High and Low Risk Types	L2 Protein	AAP20600; CBY85555; ACG75892; CBM42037; NP_040316; ACS92690; CAF05697; CAF05788; ACX32383; ACX32375, ACX32367; ACX32361; ACX32355; AAF00067; ACL12349; ACL12340; ACL12332; CAA63878; CAA63886; BAA90741; ADH94049; ADC35722; AAV91690; ABP99894; ACV88632; AAA79462; AEI61812; AEI61596; AEI61420; AEI61252; AEI61076; NP_043449; AAO15453; AAK28455; AAA79497; ABO76885; ABO76815; CCB84763; YP_002647037; AAA47055; CAA52599; CAA52594; CAA52577; CAA52571; CAA52534; NP_041786; AAC54856; CAA52589; and AAK01851.
HPV Type 16	L1 Protein	ACF77101; ABD91874; AAM74159; ACG75893; ACS92699; ACN91181; AAO85415; AEA76067; ABL84339; AAO19439; ABF06542; AAZ81569; ADB97228; ACV84005; AEF33108; ADP24270; and ABW95027.
HPV Type 18	L1 Protein	AAP20601; AEA76072; ACF37312; ADC35715; AAY57807; AAZ03393; ADP24282; ABW95029; ABF29405 and ADB96254.
